# Cholera host response gene networks: preliminary studies

**DOI:** 10.6026/97320630013347

**Published:** 2017-10-31

**Authors:** Paul Shapshak, Charurut Somboonwit, Shu Cao, John T. Sinnott

**Affiliations:** 1 Division of Infectious Diseases and International Medicine, Department of Internal Medicine, Morsani College of Medicine, Tampa, FL 33606 USA; 2 Clinical Research Unit, Hillsborough Health Department, Tampa, Florida 33602

**Keywords:** Cholera, gene networks, in vitro, in vivo, vaccines

## Abstract

Gene network analysis was performed based on published literature describing genes that are possibly interconnected with cholera vaccine responses in vitro in cell culture and in vivo in human patient punch biopsies as well as DNA extracted from blood. These studies produced divergent results. The differences should be replicated and studied further. Included in such studies, patient ethnicities, states of stress, nutrition, and health, as well as the precise characteristics of the various cholera vaccines and modes of delivery need to be considered as well.

## Background

Previously, in silico studies of Vibrio cholerae cholera toxin (CT) three-dimensional structure were performed. CT is a heterohexamer (AB5) complex composed of one subunit A (CTA) bound to a pentamer of subunit B (CTB) [1]. The current article, however, addresses the problematic issue of cholera infection from the vantage point of deciphering any gene expression networks that result from exposure of cells to cholera, in vitro and in vivo, based on published literature findings. The study of gene networks helps in understanding host cell molecular processes in infected diseased individuals, in order to characterize the impact of cholera and vaccines on host cell gene expression. We use published literature results to portray gene expression networks.

## Methodology

The GenePro Qiagen program of SABiosciences was used to produce gene networks and genes were further identified using published URL websites [2, 3, 4].

## Results

Twenty-three genes were used as input for the GenePro URL at Qiagen-SABiosciences [2, 5]. These genes include growth regulated oncogene (Gro)-a, Gro-b, Gro-g, macrophage inflammatory protein (MIP)-3a, TNF-a, leukemia inhibitory factor (LIF), macrophage inhibitory cytokine 1 (MIC-1), fibroblast growth factor 5 (FGF-5), IL-5, Urokinase receptor, Ephrin A1, IL- 8, EphA2, Dtr (HB-EGF), H2B histone family marker Q, dual specific phosphatase 5, serine threonine protein kinase (STPK), differentiation inducing factor, claudin 4, jun B proto-oncogene, nuclear receptor DNA binding protein, cytochrome P450, tubulin a3, and chloride intracellular channel 1. The analytical results show various types of interactions among the genes. In the figures, line-colors and various interactions among the genes are color-coded: red = Down-regulation, green = Up-regulation, beige = Regulation, purple = Co-expression, brown = Physical Interaction, turquoise dotted = Predicted Protein Interaction, and mauve dotted = Predicted Transcription Factor Regulation.

## Discussion

**Figure 1:**
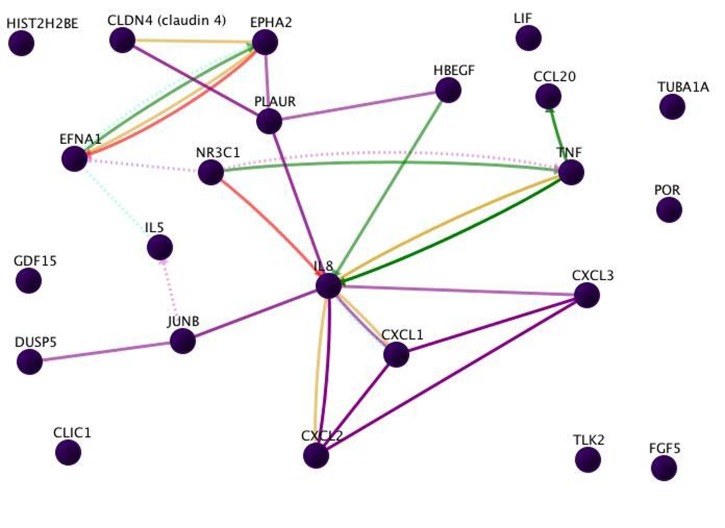
The interrelationships that are identified for the input genes. This figure shows the interactions of the 23 genes whose expression was perturbed by Cholera infection of intestinal epithelial cells in vitro in culture (2, 5). 15 of these genes show 29 direct interactions. In particular, TNF shows six interactions, IL8 shows 11 interactions, and CXCL2, CXCL2, CXCL3, with TNF show an associated set of interactions. HIST2H2BE, FGF5, TLK2, TUBA1A, POR, and CLIC1 show no interactions with the input genes.

**Figure 2:**
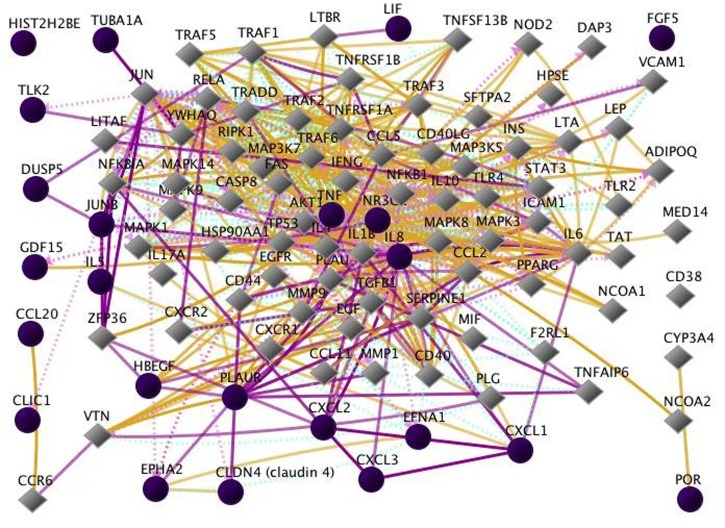
The networks of additional genes interactive with the input genes. This figure shows interactions among the 23 input genes that include the 29 direct interactions as well as additional genes and interactions. There are 67 additional genes resulting in more than 200 interactions. For improved clarity, the Down-regulation and Up-regulation interactions are not shown because their sheer number would obscure the other interactions. Interestingly, HIST2H2BE and FGF5 still show no interactions with any other genes in this more extensive diagram.

In order to treat a complex and lethal disease such as Cholera, host gene expression requires analysis to more fully understand disease pathogenesis. In prior studies [5], Vibrio cholerae vaccine strains with varying virulence (395, N16961, CVD101, CVD103- HgR, CVD110, CVD112, JBK70, and 1074-78) were used to study transcription in human intestinal epithelial cells (T84). Genes, whose expression was reproducibly induced and repressed among various strains, were identified and included inflammatory cytokines, mucosal immunity, cellular proliferation, and intracellular signaling. Virulence, thus, may be associated with variations in expression patterns, not expression of a specific gene in the human host. These genes include growth regulated oncogene (Gro)-a, Gro-b, Gro-g, macrophage inflammatory protein (MIP)-3a, TNF-a, leukemia inhibitory factor (LIF), macrophage inhibitory cytokine 1 (MIC-1), fibroblast growth factor 5 (FGF-5), IL-5, Urokinase receptor, Ephrin A1, IL- 8, EphA2, Dtr (HB-EGF), H2B histone family marker Q, dual specific phosphatase 5, serine threonine protein kinase (STPK), differentiation inducing factor, claudin 4, jun B proto-oncogene, nuclear receptor DNA binding protein, cytochrome P450, tubulin a3, and chloride intracellular channel 1. Several select genes were mapped into networks using the Qiagen Gene Pro, as shown in Figure 1 and 2. In addition to the 23 genes already identified [2, 5], the genes whose expressions are additionally perturbed include transcription, inflammation, as well as cell growth (Figure 2).

**Figure 3:**
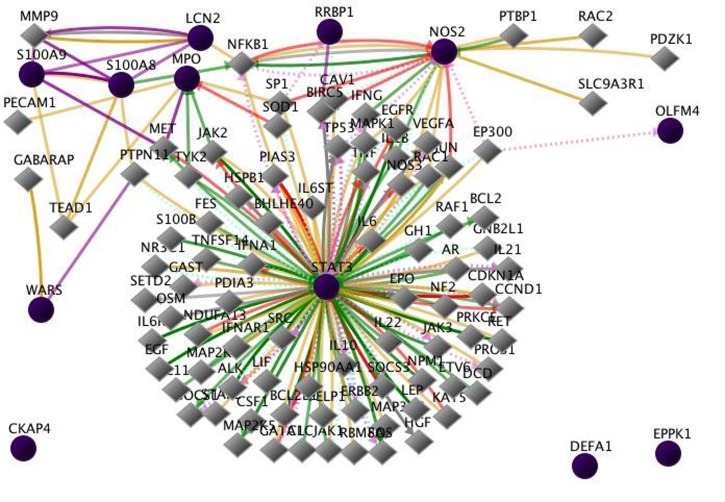
Twelve proteins were induced during acute cholera infection in duodenal tissue punch biopsies. These genes included LCN2 - Neutrophil gelatinase-associated lipocalin, WARS - Tryptophanyl-tRNA cytoplasmic synthetase, DEFA1 - Neutrophil defensin, S100A8 - Protein S100-A8, NOS2 - inducible Nitric oxide synthase, MPO - Myeloperoxidase, RRBP1 - Ribosome-binding protein 1, S100A9 Protein, OLFM4 - Olfactomedin-4, EPPK1 - Epiplakin, CKAP4 - Cytoskeleton-associated protein 4, STAT3 - Signal transducer and activator of transcription 3 [6]. This figure illustrates the interrelationships that are identified for the input genes. This figure shows the interactions of the 12 [2, 6]. In this figure, CKAP4, DEFA1, and EPPK1 show no interactions among the input genes.

**Figure 4:**
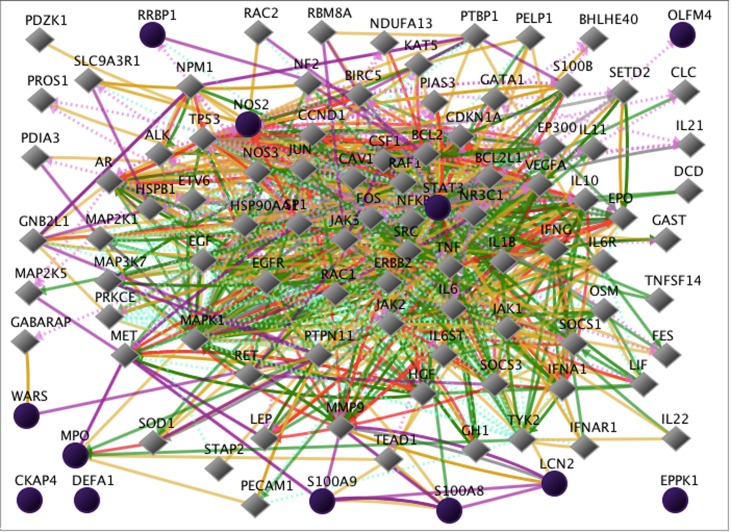
The networks of additional genes interactive with the 12 input genes of Figure 3. CKAP4, DEFA1, and EPPK1 still show no interactions with any other genes in this diagram.

However, it is important to note (Figure 3 and 4) that in vivo studies did not produce similar gene expression networks compared to the in vitro cell culture findings. A different set of twelve proteins was induced, in vivo, during acute cholera infection in duodenal tissue punch biopsies as described above. These gene were in the following categories: apoptosis, lipid biosynthesis and metabolism, cell adhesion, innate immune response, infection defense response, cytokine production, NF-kB regulation, signal transduction, cell-cell signaling, and cell spreading and migration, to name several [6].

**Figure 5:**
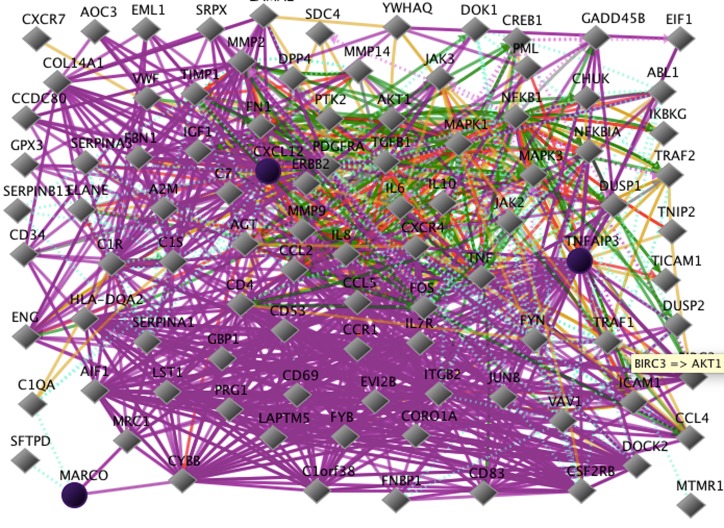
Networks of three genes that were associated with cholera vaccination response, based on human subject SNP analysis of DNA from blood in vivo. The genes are Marco, TNFAIP3, and CXCL12. These genes with additional protein interactions are shown in this figure. Marco, TNFAIP3, and CXCL12are associated with the categories of epithelial barrier integrity, scavenger receptor family, intestinal homeostasis, and leukocyte attractant/recruitment [2, 7]

The gene sets based on DNA from blood are different from the cell culture and tissue biopsy gene results (Figure 5). Methods of analysis, sampling modes, as well as medical histories impact on the interpretations of the outcomes and should be considered and analyzed.

## Conclusion

Recent cholera host gene expression studies show a divergence among results of in vitro studies vs. in vivo biopsy studies, as well as SNP analysis of DNA from vaccine blood. These differences necessitate further study and analysis as well as replication of the respective findings. Moreover, the ethnicities, states of stress, nutrition, and health of the human subjects, as well as the precise characteristics of the various cholera vaccines and modes of delivery need to be taken into account as well. Finally, the pleiotropic nature of the genes themselves may play a role in differences in gene expression.

It is relevant to ascertain the gene networks in the host that are components of the response to cholera challenge as identification of gene networks will assist in establishing areas of prevention that vaccines impact. Future manipulation of host response gene networks may increase resistance to cholera as well as improve the host response to cholera infection. Moreover, gene expression manipulation may facilitate and optimize individual response to cholera vaccines as needed. This will improve health care, save thousands of lives, as well as reduce the social and financial burden.

## Conflicts of Interest

The authors report no conflicts of interest.
